# Coexistence of nail lichen planus and lichen planus pigmentosus*

**DOI:** 10.1590/abd1806-4841.20164635

**Published:** 2016

**Authors:** Luciana Rodino Lemes, Renata Brandão Villa Verde, Sandra Maria Barbosa Durães, Adolpho de Alencar Araripe Junior, Luciana Pantaleão

**Affiliations:** 1 Universidade Federal Fluminense (UFF) - Niterói (RJ), Brazil

**Keywords:** Face, Lichen planus, Nails

## Abstract

We describe a fifty-six-year old, Afro-descendent female patient showing dystrophy of her twenty nails and hyperchromic, asymptomatic macule on her face. Histopathological examination of the macule showed vacuolization of the basal layer, melanophages in the superficial dermis and lymphoplasmocytic inflammatory infiltrate. Nail biopsy revealed orthokeratotic hyperkeratosis and lichenoid inflammatory infiltrate. Lichen planus pigmentosus is an uncommon variety of lichen planus. It is characterized by typical hyperpigmented macules on the face and neck. Nail changes might be present in 10% of lichen planus cases, but no associations with lichen planus pigmentosus have been described. We report a case of lichen planus in twenty nails associated with lichen planus pigmentosus on the patient's face.

## INTRODUCTION

Nail lichen planus and lichen planus pigmentosus are unusual variants of lichen planus (LP). Nail LP may occur alone or together with typical LP symptoms in 10% of with skin or mucosal lesions.^1,2^ Its clinical presentation ranges from nail bed dystrophy, onycholysis, and longitudinal striations to dorsal pterygium.^3^ LP pigmentosus has been described in association with lichen planus pilaris on the scalp.^4^ It shows as brown-grayish macules with a usually diffuse and symmetrical pigmentation pattern on sun-exposed areas of the face, neck and flexures. Immunological mechanisms associated with cellular immunity and exposure to ultraviolet light may be involved in its pathogenesis.^5,6^ Both variants have histopathology compatible with LP, with characteristic features. The nail LP shows more evident hyperkeratosis; the pigmented LP, in turn, shows atrophy of the epidermis and pigmentary incontinence.

## CASE REPORT

**Figure 1:**
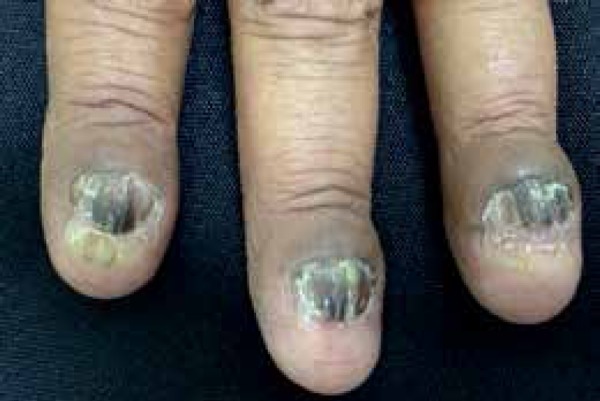
Detail of nail dystrophy of the fingers, with longitudi - nal grooves and melanonychia

**Figure 2:**
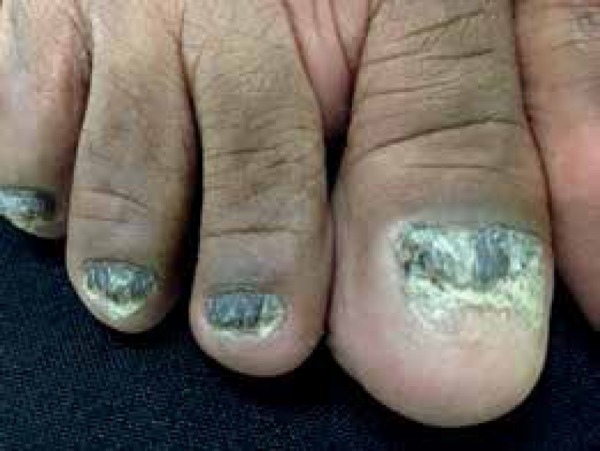
Nail dystrophy of the toes, with thickening, longitudinal striations, and melanonychia

**Figure 3:**
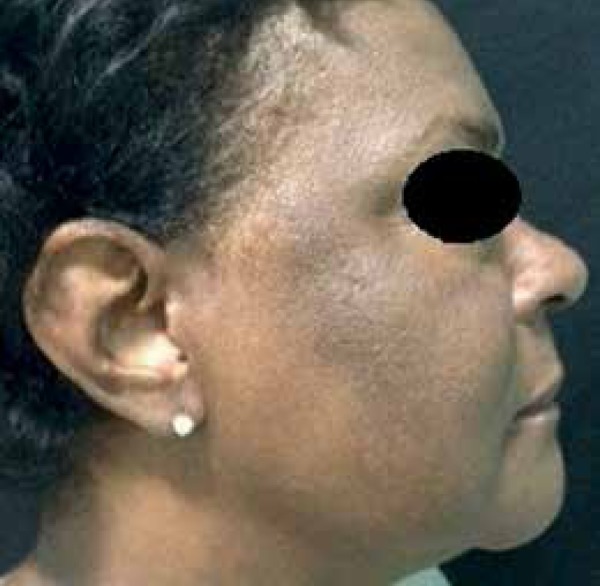
Hyperchromic macules in the zygomatic, malar, and temporal regions with mask-like appearance

**Figure 4:**
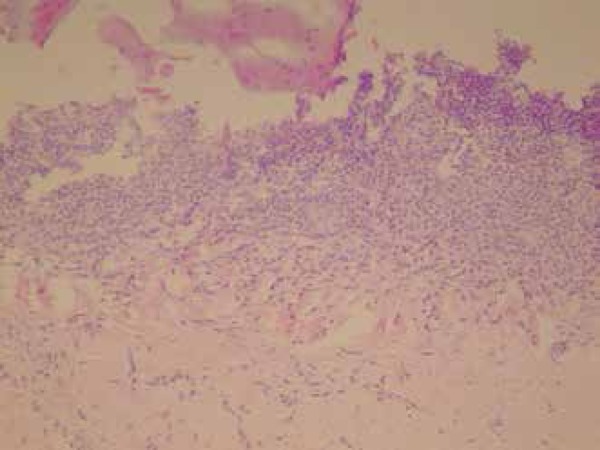
Nail biopsy showing orthokeratotic hyperkeratosis, proliferation of small vessels, and band-like lymphocytic inflammatory infiltrate

**Figure 5:**
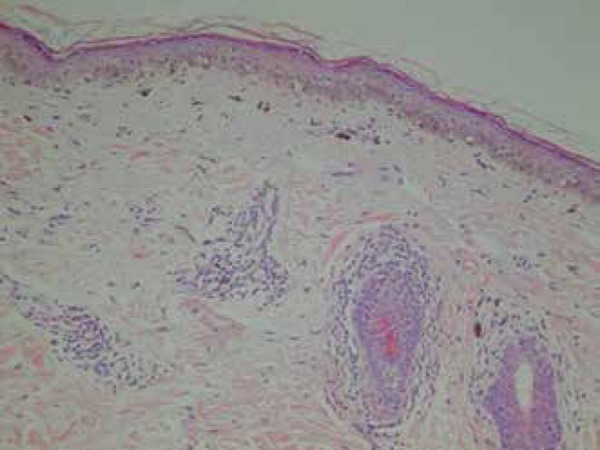
Facial biopsy showing atrophic epidermis with vacuolization of the basal layer, band-like lymphocytic inflammatory infiltrate around the follicle and melanophages in the dermis

A 56-year-old black female patient reported a history of dystrophy of her 20 nails for about two years and the emergence of hyperchromic macule, leaving her face with a mask appearance, during the same period. No itching, systemic symptoms, or other skin lesions were detected. She had a history of hypertension and labyrinthitis and had been prescribed chlorthalidone, hydrochlorothiazide, and ginkgo biloba. A dermatological exam revealed a nail dystrophy of her 20 nails associated with melanonychia, thickening of the nail plates, longitudinal streaks, and mask-like hyperchromic macules on her face (Figures 1 to 3). No signs of other skin or mucosal lesions were found. Blood count, biochemistry, liver function and thyroid hormones were within the normal range. Serology for hepatitis B and C were negative. Anatomopathological examinations of skin and nail lesions showed features consistent with LP. The nails showed orthokeratotic hyperkeratosis, proliferation of small vessels, and band-like lymphocytic inflammatory infiltrate, with no evidence of fungal structures by PAS staining (Figure 4). Biopsy of the face showed atrophic epidermis with vacuolization of the basal layer, band-like lymphocytic inflammatory infiltrate around the follicle and melanophages in the dermis (Figure 5). Based on the clinical history and histopathological examination, we diagnosed nail LP on the 20 nails with pigmented LP on the face. The treatment started with urea cream 40% for three weeks, followed by clobetasol 8% nail lacquer three times a week on the nails, and clobetasol cream once a day for 15 days on the face followed by tacrolimus 0.1% ointment once a day. The condition evolved with partial improvement of lesions.

## DISCUSSION

LP pigmentosus is a rare variant of LP. It was first described by Butani *et al.* in 1974. It is common in individuals with skin types III and IV, with a slight predominance in females in their third or fourth decades of life. It is described as brown-grayish macules on the sun-exposed areas of the face, neck, and flexures, usually without previous erythema. The lesions often evolve to diffuse or reticular pigmentation.^7-9^ It shows bilateral and symmetrical distribution - with cases of zoster-like distribution - and less commonly affect the oral mucosa. The most common pigmentation pattern is diffuse, but less common patterns might be found, such as reticular, linear unilateral, and perifolicular.^10^ Its cause remains unknown, but immunological mechanisms associated with cellular immunity and exposure to ultraviolet light appear to be involved.^5,6^ The coexistence of classic LP lesions is described in approximately 20% of patients, but reports on their association with nail LP are not common. There have been cases of LP pigmentosus associated with lichen planopilaris on the scalp.^4^ The main triggering factors described are hepatitis C infection and hepatitis B vaccine. The hepatitis C virus has been more associated with oral LP, but it is difficult to establish this relationship with LP pigmentosus, given the high prevalence of hepatitis C in the populations studied.

LP pigmentosus histopathology is characterized by epidermal atrophy with vacuolization of the basal layer, lymphohistiocytic infiltrate with a lichenoid pattern in the dermis, and pigmentary incontinence. Nail LP shows evident hyperkeratosis, in addition to the classic LP characteristics.

The main differential diagnosis of LP pigmentosus is *erythema dyschromicum perstans* (EDP), or ashy dermatosis.^6^ Early EDP injuries show an inflammatory phase characterized by erythema around the hyperchromic macules, which is not observed in the LP pigmentosus. Regarding its histopathology, the two conditions differ in their location of melanin deposit. In the EDP, melanin is located in the deeper dermis, causing a bluish-gray color, while in the LP pigmentosus, the melanin deposit is found in the superficial dermis. Other possible differential diagnoses are: LP inversus, lichenoid eruption, post-inflammatory hyperpigmentation, and fixed drug eruption. Nail LP's main differential diagnosis is psoriasis. These onychopathies are often indistinguishable, requiring histopathology to highlight the characteristics of the two diseases.

The clinical course of the LP pigmentosus has not been fully established. There may be spontaneous involution in weeks, or it might persist for years, despite treatment. Topical treatments might involve high-potency corticosteroids, tacrolimus 0.1%, and systemic treatment with immunosuppressants and hydroxychloroquine. However, none has shown favorable response in the evolution of disease.^6^

We described the case of nail LP associated with LP pigmentosus for the rare combination of the two entities.
